# Women and the Royal College of Surgeons

**Published:** 1908-10-24

**Authors:** 


### WOMEN AND THE ROYAL COLLEGE OF SURGEONS.

At the last quarterly meeting of the Council of the Royal College of Surgeons of England, held on October 15, the question of the admission of women to the diplomas of the College was further considered, and the following resolutions were adopted:—"(*a*) That steps be forthwith taken to admit women to the examinations of the Conjoint Examining Board in England and to the examination for the diploma in public health. (*b*) That women be admitted to the examinations for the Fellowship and to the examinations for the license in dental surgery." A committee was then appointed to prepare a formula for the necessary alterations of the by-laws; and it is to be noted further that it will be necessary that the revised by-laws shall be approved by the Privy Council and the Home Secretary before they can come into force.

Thus, after twelve or more years of agitation, deliberation, and discussion the diplomas of the English Conjoint Board and the coveted Fellowship of the Royal College of Surgeons of England are to be placed within the reach of women students and practitioners of medicine. We have frequently considered the claims of women to equality of treatment in this respect, and on several occasions during the present year have expressed our opinion that, in the interests of all concerned, it would be desirable to throw these diplomas open to both sexes. As is well known, the Councils of both the Royal Colleges indicated last year that each was willing to admit women to their diplomas, and formal communications and inquiries were exchanged between the two Councils. During the early part of the present year it will be remembered that the Council of the Royal College of Surgeons, although it had power to act in the matter on its own initiative, decided to take a poll of its members and Fellows upon the subject in June; intimating at the same time that it would not necessarily be bound to accept the opinion of the majority. The result of the voting was a majority of 415 against the admission of women to the membership, and a majority of 1,182 against their admission to the Fellowship. The votes of the Fellows taken separately showed a majority of 288 in favour of admitting women to the membership, and a majority of 34 in favour of admitting them to the Fellowship. The votes of the members taken separately showed a majority of 703 against admitting women to the membership, and a majority of 1,216 against admitting them to the Fellowship. In view of the facts that the Fellows approved the proposal, that the majority of members against it was comparatively small, and that only 8,543 members out of 13,800 voted, and 1,043 Fellows out of 1,373, the Council came to the conclusion on October 15 that it would be justified in admitting women to the examinations, and this decision is, of course, binding upon the members.

We think that the majority of the profession will agree that the Council has decided rightly. The English Conjoint diplomas must eventually become accessible to women, and it would be foolish to defer the inevitable. Whether the inclusion of women among the body of Fellows will be productive of benefit to the College itself or to the members thereof, is another matter. It has been suggested that last week's decision will have far-reaching effects, and that the presence of women amongst both classes of the College, amongst the rulers and the ruled, will materially influence the relative positions of members and Fellows. On the whole, this seems to us to be unlikely—at any rate for a considerable length of time. We imagine that women medical practitioners for some years to come will have other work, of greater value and importance, to perform before they attempt to interfere with the constitution of the licensing bodies which have taken them into their folds.

